# Adiposomes from Obese-Diabetic Individuals Promote Endothelial Dysfunction and Loss of Surface Caveolae

**DOI:** 10.3390/cells12202453

**Published:** 2023-10-15

**Authors:** Imaduddin Mirza, Mohamed Haloul, Chandra Hassan, Mario Masrur, Amro Mostafa, Francesco M. Bianco, Mohamed M. Ali, Richard D. Minshall, Abeer M. Mahmoud

**Affiliations:** 1 Department of Medicine, Division of Endocrinology, Diabetes, and Metabolism, College of Medicine, University of Illinois at Chicago, Chicago, IL 60612, USA; mmirza24@uic.edu (I.M.); halou57@uic.edu (M.H.); 2 Department of Surgery, College of Medicine, University of Illinois at Chicago, Chicago, IL 60612, USA; chandrar@uic.edu (C.H.); mmasrur@uic.edu (M.M.); biancofm@uic.edu (F.M.B.); rminshall@uic.edu (R.D.M.); 3 Departments of Anesthesiology and Pharmacology, College of Medicine, University of Illinois at Chicago, Chicago, IL 60612, USA; amost2@uic.edu; 4 School of Business and Non-Profit Management, North Park University, Chicago, IL 60625, USA; mali37@uic.edu; 5 Department of Kinesiology and Nutrition, College of Applied Health Sciences, University of Illinois at Chicago, Chicago, IL 60612, USA

**Keywords:** extracellular vesicles, adiposomes, glycosphingolipids (GSLs), endothelial dysfunction, caveolae, Src kinase, caveolin-1 (cav-1), endothelial nitric oxide synthase (eNOS), shear stress, flow-induced dilation (FID)

## Abstract

Glycosphingolipids (GSLs) are products of lipid glycosylation that have been implicated in the development of cardiovascular diseases. In diabetes, the adipocyte microenvironment is characterized by hyperglycemia and inflammation, resulting in high levels of GSLs. Therefore, we sought to assess the GSL content in extracellular vesicles derived from the adipose tissues (adiposomes) of obese-diabetic (OB-T2D) subjects and their impact on endothelial cell function. To this end, endothelial cells were exposed to adiposomes isolated from OB-T2D versus healthy subjects. Cells were assessed for caveolar integrity and related signaling, such as Src-kinase and caveolin-1 (cav-1) phosphorylation, and functional pathways, such as endothelial nitric oxide synthase (eNOS) activity. Compared with adiposomes from healthy subjects, OB-T2D adiposomes had higher levels of GSLs, especially LacCer and GM3; they promoted cav-1 phosphorylation coupled to an obvious loss of endothelial surface caveolae and induced eNOS-uncoupling, peroxynitrite generation, and cav-1 nitrosylation. These effects were abolished by Src kinase inhibition and were not observed in GSL-depleted adiposomes. At the functional levels, OB-T2D adiposomes reduced nitric oxide production, shear response, and albumin intake in endothelial cells and impaired flow-induced dilation in healthy arterioles. In conclusion, OB-T2D adiposomes carried a detrimental GSL cargo that disturbed endothelial caveolae and the associated signaling.

## 1. Introduction

Cardiovascular disease (CVD) is higher by up to tenfold in obese, diabetic adults than in the age-matched population [1]. Unlocking the intricate relationship between metabolic diseases and vascular dysfunction through the study of intermediate molecular events is therefore of great importance. It is widely acknowledged that fat depots are important secretory organs that release numerous bioactive molecules and that a significant portion of this secretome is found in adipocyte-derived extracellular vesicles (adiposomes) [2,3,4]. In the current study, we propose a novel role for adiposomes as a conveyor of the adipocyte-dysregulated metabolites to endothelial cells, causing endothelial dysfunction. Adiposomes are lipid envelopes that carry genetic materials, proteins, lipids, and other metabolites [5], and their production increases in metabolic diseases [6,7], contributing to inflammation and insulin resistance [8,9]. Yet, mechanisms of adiposome interaction with endothelial cells have remained to be explored. Therefore, this study will pave the way to examine biological messages that can be transferred by adiposomes to endothelial cells. In addition, it will reveal some of the effects of these adiposomes on endothelial cell structure and function.

Obesity and insulin resistance are characterized by changes in sphingolipid metabolism and an induced synthesis of glycosphingolipids (GSLs), which may provide a common pathway linking excess nutrients and inflammation to increased cardiovascular risk [10]. GSLs are a family of active lipids generated via the glycosylation of ceramide [11]. The generation of GSLs is augmented in inflamed tissues and is driven by the local milieu of high glucose and inflammation-mediated induction of the activity of GSL synthesis enzymes [12,13]. High levels of GSLs were shown to be associated with obesity, diabetes, and cardiovascular diseases [14,15]. GSLs were reported to promote atherosclerosis, retinopathy, and neuropathy in diabetic models [16,17,18]. Furthermore, the content of lactosyl ceramides in atherosclerotic lesions was found to be 10- to 50-fold higher than the low-density lipoproteins [19]. However, the critical barrier to progress in this area is that the method of delivery of excess GSLs to endothelial cells and the precise mechanism that mediates their impact on vascular function are poorly understood. GSLs are among the most deleterious lipid classes since they induce aberrant signaling when deposited in cells incompatible with lipid storage, such as endothelial cells.

Previous studies reported the tendency of GSLs to accumulate in surface caveolae [20]. The latter are 50 to 100 nm plasma membrane invaginations abundant in endothelial cells and critical for lipid homeostasis, vesicular trafficking, and signal transduction [21]. In endothelial cells, caveolae cover more than 50% of the cell surface [21]. The major protein component in the caveolar bulb is caveolin-1 (cav-1). Cav-1 and other caveolar proteins represent a docking site for endothelial nitric oxide synthase (eNOS) and other proteins involved in angiogenesis, atherogenesis, adhesion, junctional permeability, and signal transduction, such as Src kinase [21]. Cav-1 has been identified as a Src kinase substrate [22]. Tyrosine 14 (Y14) in cav-1 is phosphorylated by Src kinase and has been linked to surface caveolae fission and internalization [23,24]. Accordingly, interfering with Src kinase activation inhibited cav-1 phosphorylation and caveolar internalization in endothelial cells [25]. Interestingly, prolonged cav-1 phosphorylation leads to polyubiquitination and degradation, which is thought to contribute to caveolar reduction on the endothelial cell surface [26]. 

Previous research by our team reported a caveolar loss from the endothelial cell surface in response to Src activation and reciprocal regulation of cav-1 and eNOS in endothelial cells. These studies showed induced phosphorylation of eNOS in response to caveolar loss and subsequently detrimental effects on endothelial cell function, such as oxidative stress and nitrosylation of critical endothelial proteins [27]. In the current study, we sought to examine the lipid content of adiposomes produced by obese diabetic individuals versus lean, healthy controls. Also, we sought to investigate the effect of obese diabetic adiposomes on (1) endothelial caveolae and the associated signaling and (2) on vasoreactivity of adipose tissue arterioles isolated from lean, healthy individuals. 

Adiposome production by metabolically challenged adipocytes is a quality control mechanism for maintaining cellular homeostasis and eliminating unrequired metabolites such as GSLs [28]. This spillover of lipid metabolites into the circulation is followed by ectopic deposition in cells incompatible with lipid storage, such as endothelial cells [29]. Unlike the protein and nucleic acid cargo, the lipid content in extracellular vesicles and its implication in vascular function has been far less explored. Accordingly, the current research may contribute to a better knowledge of new risk factors for the development of CVD in obese diabetic patients, as well as new therapeutic targets.

## 2. Materials and Methods

### 2.1. Study Subjects

Adipose tissue (AT) biopsies were obtained at the University of Illinois Hospital from 15 obese-diabetic subjects (body mass index (BMI) higher than 30 kg/m^2^) and 15 non-obese controls (BMI less than 25 kg/m^2^) during bariatric and elective surgeries. Adults below 50 years of age were recruited to eliminate potential detrimental effects of aging on arterial function, accelerated after 50 years of age. The criteria for T2D were confirmed use of diabetes medication and a fasting glucose level of ≥126 mg/dL. Exclusion criteria included current smoking, pregnancy, prior metabolic surgery, current liver, kidney, or heart disease, malignancy, and acute or chronic inflammatory diseases. The methodologies and procedures employed in this study were approved by the Institutional Review Board of the University of Illinois (Protocol # 2017-1125) and were found to be in compliance with the latest version of the Declaration of Helsinki.

### 2.2. Adipose Tissue Processing and Endothelial Cell and Adiposome Isolation 

Surgically obtained fresh AT biopsies were washed in sterile Medium 199 (M199) (Gibco, Waltham, MA, USA) and minced into small pieces around 5–10 mg each (1–2 mm^3^), using a sterile scalpel and sharp scissors. Collagenase solution (2–3 mL per 1 g of AT) that consists of 1 mg/mL Type 1 collagenase (Worthington) and 4% BSA in M199 medium was added to the minced AT followed by incubation for 15 min in a cell culture incubator. Tissues were then incubated in a 37 °C water bath for 60 min with frequent shaking. The digested AT were filtered through a nylon mesh filter and centrifuged at a low speed (500× *g* for 1 min) [30]. Floating adipocytes were collected, washed three times, and cultured in M199 medium with 5% exosome-free fetal bovine serum (FBS) and 1% penicillin/streptomycin for 48–72 h. Cells were cultured with and without the glucosylceramide synthase inhibitor (1R,2R)-nonanoic acid [2-(2′,3′-dihydro-benzo[1,4]dioxin-6′-yl)-2-hydroxy-1-pyrrolidin-1-ylmethyl-ethyl]-amide-l-tartaric acid salt (Genz-123346; 1 μmol/L) purchased from Sigma-Aldrich (St. Louis, MO, USA). Culture media were centrifuged at 4 °C at 1000× *g* for 5 min, followed by centrifugation at 15,000× *g* for 15 min. The resulting supernatant was then filtered through a 0.45 μm syringe-driven filter to remove cellular debris, apoptotic bodies, and microvesicles. Filtered samples were then ultracentrifuged at 150,000× *g* for 2 h to pellet adiposomes. Adiposomes were then resuspended in PBS and measured using nanoparticle tracking analysis (NTA) to evaluate particle size and concentration. NTA was carried out with a NanoSight NS300 Analyzer (Malvern Instruments Ltd., Malvern, UK), software version 2.3, screen gain 4, and camera level 10. Five 1 min movies were produced for each sample, with the analysis screen gain set to 10 and the detection threshold set to 4 [31].

Following AT digestion, the stromal vascular fraction (SVF) was used to isolate endothelial cells. Red blood cell lysis buffer was used to purify the SVF for better binding to magnetic beads. Each sample was resuspended in an appropriate amount of CD31 human MicroBeads (human, Miltenyi Biotec, Cat#130-091-935) and FcR Blocking Reagent based on the provided protocol by the manufacturer. Samples were then incubated for 30 min at a cold temperature (4 °C) followed by centrifugation at 300× *g* for 5 min, repeated washing steps, and finally, cells were resuspended in the provided washing buffer. Samples were loaded in the LS columns (Miltenyi Biotec, Gaithersburg, MD, USA) that were placed in the magnetic field of MACS MultiStand (Miltenyi Biotec) equipped with QuadroMACS™ Separator (Miltenyi Biotec). Columns were then washed 3 to 4 times, removed from the magnetic separator, and placed in a 15 mL falcon tube. CD31-positive endothelial cells were then flushed out of the column using an appropriate volume of washing buffer.

### 2.3. Endothelial Cell Culture and Treatment

Human adipose microvascular endothelial cells (HAMECs) were obtained from Lonza Walkersville Inc. (Walkersville, MD, USA), and passages 3 through 6 were used in the following experiments. Cells were maintained in ECM media with L-glutamine supplemented with 5% fetal bovine serum (FBS) and 1% penicillin/streptomycin. Experiments were performed using passages from 2 to 6. All cell culture media were purchased from ScienCell Research Laboratories (Carlsbad, CA, USA). Cells were cultured in a CO_2_ incubator at 37 °C. Cells cultured in 35 mm dishes were treated with adiposome particles (5 × 10^11^) or GM3 (500 nmol/L) for periods that ranged from 30 min to 24 h with and without the Src kinase inhibitor, PP2 (4-amino-5-(4-chlorophenyl)-7-(t-butyl)pyrazolo[3,4-d]pyrimidine; 1 µmol/L) purchased from Sigma-Aldrich (St. Louis, MO, USA). All experiments were conducted in triplicates, and the findings were expressed as the mean ± standard error (SE).

### 2.4. Isolation of Endothelial Cell Total Cell Lysate and Cytoplasmic/Membranous Protein Fractions

The extraction of total cell lysate was performed using RIPA lysis buffer, which is composed of various components including 150 mM NaCl, 20 mM Tris-HCl (pH 7.5), 1 mM EGTA, 1 mM Na2 EDTA, 1% NP-40, 2.5 mM sodium pyrophosphate, 1% sodium deoxycholate, 1 mM Na3VO4, 1 mM b-glycerophosphate, and 1 µg/mL leupeptin (Cell Signaling, Danvers, MA, USA). Additionally, the lysis buffer was supplemented with a protease and phosphatase inhibitor mixture (MS-SAFE) obtained from Sigma-Aldrich (St. Louis, MO, USA). Membranous and cytoplasmic protein fractions were isolated via a Subcellular Protein Fractionation Kit (ThermoFisher Scientific, Waltham, MA, USA). Briefly, the cell pellet was washed and left to dry, followed by adding the membranous and cytoplasmic extraction buffers following the recommended volume and centrifugation steps in the kit’s manual. First, the cytoplasmic extraction buffer was added and incubated at 4 °C for 10 min, followed by centrifugation at 500× *g* for 5 min and transfer of the cytoplasmic supernatant to a clean tube. During the second phase, the membranous extraction buffer was introduced and allowed to incubate at a temperature of 4 °C for a duration of 10 min. Subsequently, centrifugation was performed at a force of 3000× *g* for a period of 5 min. The membrane extract was then carefully transferred to a clean tube.

### 2.5. Lipid Extraction and Mass Spectrometry Analysis

Lipid extraction from adiposomes was performed using the Folch method. Briefly, ice-cold methanol and chloroform (1:2, *v*/*v*) were added directly to the samples (adiposome pellet). For non-targeted lipid analysis in adiposomes, the lipid internal standards, EquiSPLASH LIPIDOMIX mixture (Avanti Polar Lipids, Birmingham, AL, USA) was used. Samples were sonicated, vortexed, and kept on ice for 30 min, followed by the addition of ice-cold chloroform/methanol/water (8:4:3, *v*/*v*/*v*) and centrifugation at 10,000 rpm for 5 min at 4 °C to induce phase separation. The lower phase (organic layer) was collected and evaporated under a nitrogen stream at 30 °C. The extracted lipids were resuspended in methanol and chloroform (1:1, *v*/*v*) and transferred to a liquid chromatography vial with a 200 μL glass insert. A quality control sample was generated by combining a portion of each individual sample. The lipid extracts were subjected to analysis using an Agilent 6545 Q-TOF LC-MS system, which was operated with the Agilent Mass Hunter acquisition software version B.08.00. The mass spectrometer was utilized in a 2 GHz extended dynamic range mode, utilizing precursor ion analysis for conducting relative quantification tests in positive ion mode. Calibration was conducted using internal references. The composition of mobile phase A consisted of a mixture of 90% water and 10% methanol (*v*/*v*), supplemented with 10 mM ammonium acetate and 0.5 mM ammonium fluoride. On the other hand, mobile phase B was composed of a mixture in a ratio of 5:3:2. The mixture consists of isopropanol, methanol, and acetonitrile in a volumetric ratio, supplemented with 10 mM ammonium acetate and 0.5 mM ammonium fluoride. The lipid extracts that had been resuspended were introduced onto a 2.1 × 100 mm Agilent Poroshell C18, 2.7 μm column (Agilent Technologies Inc., Santa Clara, CA, USA) for separation. This was achieved using an Agilent 1290 UPLC system, employing a gradient consisting of 70% B from 0 to 1 min, 86% B from 3.5 to 10 min, and 100% B from 11 to 17 min. The flow rate used was 300 μL/min. A uniform post-column equilibration duration of 5 min was employed for all experimental runs. The experimental conditions were defined by the following parameters: the temperature of the gas was set at 200 °C, the flow rate of the drying gas was maintained at 11 L/min, the nebulizer pressure was set to 35 psi, the temperature of the sheath gas was maintained at 350 °C, the flow rate of the sheath gas was set at 12 L/min, the voltage applied to the VCap was 3000 V, and the fragmentor voltage was set to 145 V. Data were gathered for the purpose of relative quantification, with a scan speed of 4 MS spectra per second. The construction of the lipid database involved the utilization of pooled samples. A repetitive MS/MS workflow was executed using the Mass Hunter acquisition software version B.08.00. This workflow was applied to numerous injections of the pooled samples, with a scan speed of 10 MS and 3 MS/MS spectra per second.

Lipid raft isolation from endothelial cells was performed using Minute™ Plasma Membrane-Derived Lipid Raft Isolation Kit (Invent Biotechnologies Inc., Plymouth, MN, USA). Lipid extraction from isolated lipid rafts was performed as described above. For targeted lipidomics in endothelial cell membranes, a mixture of C16 Lactosyl (β) Ceramide (d18:1/16:0) and C16 Ganglioside GM3-d9 (d18:1/16:0-d9) standards for LacCer and GM3 targeted analyses (Avanti Polar Lipids, Birmingham, AL, USA) was used. Five microliters of the calibrator/sample were injected into an AB SCIEX 5500 QTRAP system coupled to an Agilent 1290 UPLC system. At a flow rate of 300 L/min, all samples were eluted from an Agilent Poroshell 120 SB-AQ 2.7 m, 2.1 × 100 mm column (P/N 685775-914). The column compartment was maintained at 40 °C. LC elution began with 50% mobile phase A (0.1% FA in H_2_O) for 1 min, followed by a linear gradient increase in mobile phase B (0.1% FA in ACN) from 50% to 100% over the same period. The column was maintained at 100% B for 7 min before being re-equilibrated to its initial state of 50% A for 3 min. The autosampler temperature was kept at 4 °C. MS data were acquired by MRM scan at positive mode. ESI spray voltage and source temperature were maintained at 4.5 kV and 500 °C, respectively. Different chain length combinations of positively charged LacCer and GM3 were detected by monitoring their transition to signature product ions.

The raw LC-MS/MS data were utilized to generate a fragmentation-based (MS/MS) library. This library included *m*/*z* precursors and retention times for all lipids that were identified through the use of the Lipid Annotator software version B.07.01 (Agilent Technologies Inc., Santa Clara, CA, USA). The software was configured with specific settings, namely the inclusion of lipid species for positive ionization ([M + H]+, [M + Na]+, [M + NH_4_]+, [M + H − H_2_O]+, [M + Na − H_2_O]+, [M + NH_4_ − H_2_O]+), a Q-Score threshold of 60 or higher, and a mass deviation limit of 10 ppm or less. The LC-MS data files were subjected to processing using the Profinder program (version B.10.00, Agilent Technologies Inc., Santa Clara, CA, USA). In this study, we extracted molecular characteristics for peaks with counts ≥ 5000 in the positive mode. Specifically, we focused on ions such as [M + H]+, [M + Na]+, [M + NH_4_]+, [M + H − H_2_O]+, [M + Na − H_2_O]+, and [M + NH_4_ − H_2_O]+. To perform this extraction, we employed an isotopic model that accounted for common organic compounds without halogens. The compound list obtained was subsequently refined by applying additional filters, including a minimum absolute height of 10,000 counts, a minimum quality score of 60, and the presence of two or more isotopes in the compounds. Furthermore, the retention period for each compound was adjusted to within a range of ±0.1 min, employing a mass accuracy window of ≤5.0 parts per million (ppm). The peaks were then quantified using the Agile integrator within the Profinder program. The retention time and fragmentation matching of each integrated peak were individually evaluated. Subsequently, the processed data file was exported and subsequently imported into the Mass Profiler Professional program (version 15.1, Agilent Technologies Inc., Santa Clara, CA, USA), wherein each data set was subjected to separate analysis. The compound abundance results were subjected to baseline correction using the median abundance and afterward adjusted based on the lipid class in the internal standard (IS).

### 2.6. Biotin Switch Assay

Biotin Switch Assay was used to detect cav-1 S-nitrosylation following previously published methods [26,32]. Briefly, endothelial cells were cultured in a serum-free medium for 3 to 5 h and then treated with adiposome particles (5 × 10^11^) with and without L-NAME or PP2 for 15 min. Endothelial cells were then harvested in lysis buffer, and 25% SDS along with 10% S-Methyl methanethiosulfonate (Sigma Aldrich; St. Louis, MO, USA) were added to the cell lysates to block thiol groups. Subsequently, ascorbate was utilized to facilitate the conversion of S-nitroso (SNO) groups to free thiol groups. This was followed by protein biotinylation via biotin-HPDP (ThermoFisher Scientific, Waltham, MA, USA). The biotinylated protein was pulled down via streptavidin beads and resolved by SDS–PAGE. As loading controls, we used the same samples prior to immunoprecipitation. A similar protocol was used to test cav-1 S-nitrosylation in AT-isolated endothelial cells.

### 2.7. Western Blotting and Cav-1 Oligomerization

Thermo Fisher Scientific’s Pierce BCA Protein Assays were used to measure the total protein concentration, which was then transferred to polyvinylidene fluoride (PVDF) after electrophoresis on 4–12% Bis-Tris gradient gels. Membranes were subsequently blocked using Intercept (TBS) Blocking Buffer (LI-COR Biosciences; Lincoln, NE, USA) and incubated in primary monoclonal antibodies (total cav-1, phospho-cav-1 Tyr14, total eNOS, phospho-eNOS Ser1177, and NADPH oxidase (Nox2) (Cell Signaling, Danvers, MA, USA), total Src, phospho-Src Tyr416 (Thermo Fisher Scientific, Waltham, MA, USA) at 4 °C overnight. Following this, infrared IRDye-labeled secondary antibodies (LI-COR Biosciences) were applied for 1 h at room temperature and protected from light. The membranes were washed with TBS + 0.1% Tween-20, dried at room temperature, and imaged in the appropriate channel (700 nm for IRDye680TM antibodies, 800 nm for IRDye800TM antibodies) using an Odyssey Clx infrared imaging system [33]. The quantification of images was performed using Image Studio ver. 4.0 (LI-COR Biosciences) to determine the intensity of phosphorylated protein bands relative to the expression of their corresponding total proteins.

Low-temperature SDS-PAGE was used to detect eNOS dimers utilizing the previously reported methods [34]. Briefly, total proteins were incubated at 37 °C for 5 min in 1X Laemmli buffer without 2-mercaptoethanol. The samples were then separated on Bio-Rad 4–12% Bis-Tris gradient electrophoresis. Before electrophoresis, gels, and buffers were equilibrated at 4 °C, and during electrophoresis, the gel temperature was maintained at <15 °C by placing the buffer vessel in an ice bath. The samples were then transferred to PVDF membranes for Western blotting. For cav-1 oligomerization, endothelial cells were cultured in a serum-free medium for 3 to 4 h and treated with adiposome with and without L-NAME or PP2 for indicated times. Samples were heated at 60 °C in a loading buffer plus DTT. Finally, samples were gel electrophoresed for Western blot analysis.

### 2.8. Confocal Microscopy

For adiposome uptake assay, adiposomes were labeled with BODIPY™ 493/503 (4,4-Difluoro-1,3,5,7,8-Pentamethyl-4-Bora-3a,4a-Diaza-s-Indacene) (ThermoFisher Scientific, Waltham, MA, USA) using the manufacturer protocol. Endothelial cells were cultured in glass-bottom 35 mm dishes and treated with adiposomes for 30 to 60 min. Treated cells were washed and fixed with 4% paraformaldehyde for 15 min at room temperature, followed by washing, permeabilization with 0.01% tween 20, and blocking non-specific binding with 1% bovine serum albumin (BSA) for 1 h. Cells were then incubated with the primary monoclonal antibodies, VE-cadherin or total cav-1 (Cell Signaling, Danvers, MA, USA) diluted in 1% BSA blocking buffer for 1 h at room temperature. After washing, cells were incubated with fluorophore-conjugated secondary antibodies for 1 h at room temperature. Finally, 30 μL of VECTASHIELD^®^ Antifade Mounting Medium (Vector Laboratories, Burlingame, CA, USA) was added. The cells were enclosed by a coverslip and captured using a 63 × 1.2 N.A. objective on a Zeiss LSM 510 META microscope (Carl Zeiss MicroImaging, Oberkochen, Germany). The imaging process involved utilizing a 488-excitation laser line and adjusting the pinhole to reach 1 Airy unit.

### 2.9. Electron Microscopy

For adiposome visualization, adiposomes were fixed with 2% Paraformaldehyde for 5 min and loaded onto thin formvar/carbon film coated 200 mesh copper EM grids and immediately stained with filtered 1% uranyl acetate. Excess stain was removed, and the grid was rinsed with water, dried for 10 min at room temperature, and examined by JEM-3010 transmission electron microscope. For caveolae visualization, treated endothelial cells were detached, washed, pelleted, and then fixed in 2.5% glutaraldehyde (Sigma Aldrich, St. Louis, MO, USA) for 1 h at room temperature. Cell pellets were washed with 1X phosphate buffer saline (PBS) for 5 min, then fixed with 1% citric acid for 1 h. Following fixation, cell pellets were dehydrated in gradient ethanol (50%, 70%, 90%, and 100%) and then stained with OsO4. For penetration and polymerization, cell pellets were embedded in Epon^®^ Resin 828 (Sigma Aldrich). Ultrathin sections of 70–80 nm in thickness were obtained from the cell pellets using the Leica EM UC7 ultra-microtome, manufactured by Leica Microsystems Inc. in Buffalo Grove, IL, USA. These sections were then placed on a 100 mesh grid and subjected to a double staining procedure involving immersion in a 4% uranyl acetate solution for 3 min, followed by immersion in a 2% lead citrate solution for 20 min. The slices were examined using a JEM-3010 transmission electron microscope (JEOL USA, Inc., Peabody, MA, USA).

### 2.10. Adiposome Uptake and Lipid Fusion Assays

Adiposomes were fluorescently labeled using BODIPY TR ceramide (Life Technologies, Carlsbad, CA, USA), a red fluorescent stain with absorption/emission wavelengths of 589 and 617 nm, respectively. Briefly, 1 mmol/L stock solution of the BODIPY stain was prepared, and one μL was added to every 100 μL of adiposome samples to reach a final concentration of 10 μmol/L. Adiposomes were incubated with the BODIPY stain at 37 °C for 20 min, followed by filtration through Exosome Spin Columns (MW 3000, Life Technologies) to remove excess unbound stain. For adiposome uptake assays, HAMECs were incubated for 30 min with fluorescently labeled adiposome particles (8 × 10^9^). Cells were washed, fixed, labeled with VE-cadherin to define cell boundaries, and imaged using a Zeiss LSM 710 confocal microscope. For lipid fusion assays, adiposomes were labeled with octadecyl rhodamine B chloride (R18, ThermoFisher Scientific, Waltham, MA, USA) (20 μM, 1 h, room temperature) following previously published protocols for labeling extracellular vesicles [35]. To remove the unbound stain, labeled adiposomes were purified by gel filtration (Sephadex G-75). Ten micrograms of labeled adiposomes were then incubated with unlabeled cultured endothelial cells that were serum-starved for 2–3 h. The fusion of labeled adiposomes and unlabeled membranes is expected to dilute the probe and subsequently increase fluorescence. Therefore, 10–15 min after adiposome incubation with endothelial cells, cells were imaged using a fluorescence microscope (Carl Zeiss MicroImaging, Oberkochen, Germany) with an excitation/emission wavelength of 560/590 nm. Images were analyzed using the ImageJ Particle Analysis module.

### 2.11. Nitric Oxide and Peroxynitrite Measurements

Nitric oxide (NO) generation was assessed by measuring the levels of nitrates and nitrites (stable metabolites of NO) in the cell culture media. Cell culture media from treated cells were collected and centrifuged to remove cell debris and detached cells. Nitrates and nitrites in the cell culture media were measured using the Griess assay kit (Cayman Chemicals, Ann Arbor, MI, USA) [36]. Briefly, the assay begins by converting all nitrates into nitrites using the supplied nitrate reductase enzyme, followed by the sequential addition of Griess reagents I and II, which convert nitrites into a dark purple azo compound. The developed color intensity was then measured at 540 nm, as recommended by the manufacturer, using a microplate reader. Nitrite concentration calculations were performed using the generated nitrate standard curve. For peroxynitrite measurements, Peroxynitrite Assay (Abcam) was used. Cultured cells were pre-stained with a peroxynitrite sensor green (10×) at 37 °C for 60 min protected from light, followed by incubation with adiposomes with and without PP2 or Tetrahydrobiopterin (BH4) for the recommended periods. The fluorescence intensity was quantified using a microplate reader, with excitation and emission wavelengths set at 490 nm and 530 nm, respectively.

### 2.12. Shear Stress Experiments

Endothelial cells were cultured in fibronectin-coated ibidi 4 chamber μ-slide (ibidi USA, Inc., Fitchburg, WI, USA) at a density of 10^5^ cells/well. Cells were incubated for 8 to 10 h to allow them to adhere to the bottom of the slide. The chamber slides were subsequently interconnected sequentially within a flow configuration consisting of a peristaltic pump, pulse dampener, and requisite silicone tubing and fittings, adhering to the comprehensive procedure outlined by Lane et al. [37] in their published protocol. The cells were initially exposed to the parallel flow condition by passing media through them at a flow rate determined to provide an average shear stress of 5 dynes/cm^2^, as calculated using the following formula:

$$Q=\frac{\tau \cdot w\cdot h^{2}}{6\cdot \mu }$$

where Q represents the desired flow rate, τ represents the target shear stress acting tangentially on the cells, w represents the width of the flow chamber, h represents the height of the flow chamber, and μ represents the viscosity of the perfusate (cell culture medium). The viscosity (μ) of the medium typically employed is 0.9 centipoise (cP), which is equivalent to 0.009 g per cubic centimeter per second (g cm^−1^ s^−1^). A commonly employed range for the goal shear stress (τ) is 15–20 dynes/cm^2^, which is representative of the average arterial shear stress. The dimensions of our chamber are conventionally characterized by a width (w) of 46 mm and a height (h) ranging from 2 to 2.5 mm. The aforementioned procedure was carried out until cellular alignment was observed in the direction of fluid flow, which typically occurred within a timeframe of 4 to 6 h. After the conditioning process, the endothelial cells were exposed to shear stress at an average magnitude of 15 to 20 dynes/cm^2^ for a duration of 2 to 4 h. The flowing media contains the designated treatment (adiposomes or GM3 with and without PP2). Cells were imaged via Bright-field microscopy then they were collected for RNA isolation and analysis of gene expression.

### 2.13. Fluorescently Labeled Albumin Uptake in Endothelial Cells

For this experiment, endothelial cells were cultured on fibronectin-coated glass-bottom 35 mm dishes. Cells that were preconditioned with the designated treatments were incubated with 10 μg/mL Texas Red conjugated bovine serum albumin (BSA) in basal media containing 0.1 mg/mL unlabeled BSA. The cells underwent a process of acid washing using a buffer with a pH of 2.5, followed by a wash with Hank’s balanced salt solution (HBSS) to eliminate any surface-bound BSA. Subsequently, the cells were fixed using 4% paraformaldehyde and stained with cav-1 and DAPI. Finally, the cells were photographed using the procedures outlined in Section 2.8.

### 2.14. Measurements of Microvascular Flow-Induced Dilation and NO and ROS Production

Samples of AT were dissected in order to isolate tiny arterioles. These arterioles were subsequently cleared of any excess connective tissue and fat. As we previously reported, the internal diameter of dissected vessels was assessed in relation to a progressively escalating pressure gradient. The vessels were cannulated using glass microcapillaries that were placed into an organ perfusion chamber. The microcapillaries were secured by tying the vascular ends using nylon Ethilon monofilament. Subsequently, the organ chamber was relocated to an inverted microscope that was linked to video microscopy to observe and assess the vasoreactivity of arterioles. The terminals of the cannulated arterioles were linked to two reservoirs that contained Krebs buffer. The manipulation of the reservoirs in a symmetrical manner, involving upward and downward movements, resulted in the generation of an intraluminal pressure gradient ranging from 10 to 100 cm of water (cm H_2_O). The Krebs buffer solution was supplemented with a combination of oxygen (21%), carbon dioxide (5%), and nitrogen (74%), while its pH and temperature were upheld at 7.4 and 37 °C, respectively. After conducting baseline measurements, the cannulated vessels were subjected to constriction using endothelin (ET-1) at a concentration of 10^−6^ mol/L. Only vessels that exhibited a constriction of more than 30% were included in further measurements. The measurement of arteriolar vasoreactivity was conducted under two conditions: with the presence and absence of the endothelial nitric oxide synthase inhibitor, L-NG-Nitro arginine methyl ester (L-NAME, 10^−4^ mol/L). The calculation of vasodilation % involved the normalization of arteriolar diameter at each pressure gradient to the diameter observed after the administration of ET-1. For the Ad-cav-1 transfection experiments, a custom-designed adenoviral vector expressing cav-1 with VE-Cadherin endothelial cell-specific promoter was constructed in Vector BioLabs (Malvern, PA, USA). Isolated arterioles were intramurally transfected with the cav-1 vector (100 MOI) or an empty vector for 8 h at 37 °C following previously published protocols [38,39]. cav-1 overexpression in arterioles transfected with Ad-cav-1 versus an empty vector was verified via Western blotting techniques. The generated NO and ROS by isolated arterioles were measured as we previously described [36]. using the NO Detection Kit (Enzo Life Sciences, Farmingdale, NY, USA) and the ROS indicator (2′,7′-dichlorodihydrofluorescein diacetate (H2DCFDA). In cannulated arterioles, measurements were made in response to a pressure gradient of about 60 cm H_2_O. Arterioles underflow were excised, cleaned, and mounted on microscope slides after being treated with the NO and ROS detection reagents. At 650 and 495 nm, respectively, images were captured using a fluorescent microscope (Eclipse TE 2000, Nikon, Japan). Every experiment followed the same incubation, staining, and imaging procedures. Using Image J software version 1.8.0 (NIH, Bethesda, MD, USA), images were then examined for fluorescence intensity. The fluorescent signal was then represented in arbitrary units.

### 2.15. Statistical Analysis

The obtained data were representative of triplicates from three different experiments and were expressed as the average value ± the standard error. The data were subjected to statistical analysis using the Student’s *t*-test or one-way ANOVA, followed by a post-hoc analysis using Bonferroni’s method where deemed suitable. Statistical significance was determined by considering results with a *p*-value less than 0.05.

## 3. Results

### 3.1. Subject Characteristics

Anthropometric and cardiometabolic risk measurements of the study’s human participants are summarized in Table 1. As expected, body weight and body mass index (BMI) were significantly higher in the obese diabetic (OB-T2D) group compared with the lean healthy controls (LHC). Also, significant differences were found in diastolic blood pressure and some measurements of glucose and lipid metabolism (Table 1).

### 3.2. Characterization of Adiposomes Isolated from Human VAT

**Figure 1:**
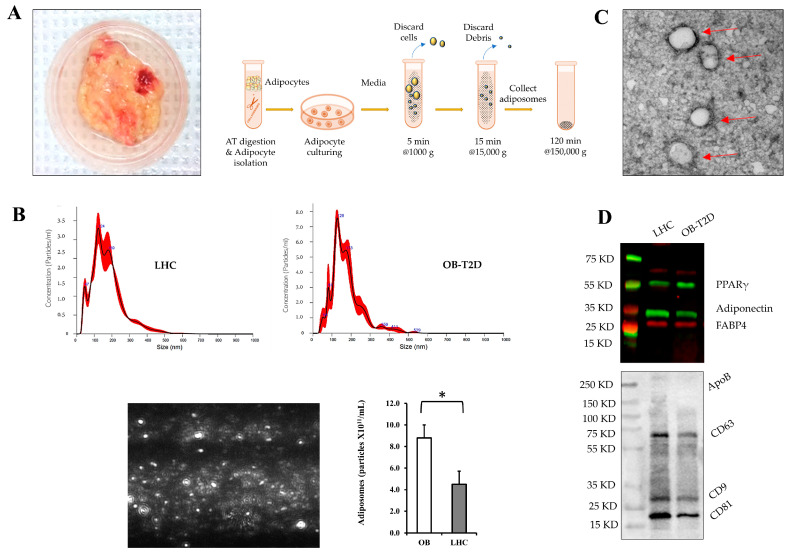
Adiposome isolation and characterization. (**A**) An illustration of AT processing and the centrifugation/purification steps required for adiposome isolation. (**B**) Representative screenshot and charts from the Nanoparticle Tracking Analysis of adiposomes using NanoSight NS300. The bar chart depicts the average number of adiposome particles isolated from OB-T2D and LHCs (*n* = 15 each). (**C**) Transmission Electron Microscopy (TEM) image of the isolated adiposomes. Red arrows point to the adiposomes (**D**) Representative Western blots for analyzing adiposome-extracted proteins for tetraspanins (CD9/DC63/CD81) and adipogenic proteins (PPARγ, adiponectin, FABP4). * *p*-value < 0.05.

Adiposomes were isolated from VAT from OB-T2D and LHCs via several steps of centrifugation and purification as described in Section 2 (Figure 1A). The isolated adiposomes were then examined for their characteristics using nanoparticle-tracking analysis (NTA). Adiposomes had diameters between 50 and 300 nm, and their average numbers were higher for the OB-T2D compared with (8.8 × 10^11^ particles/mL vs. 4.5 × 10^11^ particles/mL, *p* = 0.005) (Figure 1B). Since NTA does not distinguish between extracellular vesicles (EVs) and similarly sized molecules such as small lipoproteins, samples were visualized by transmission electron microscopy (TEM), which indicated a vesicle structure (Figure 1C). Analyzed by Western blotting, the protein content of isolated adiposomes demonstrated the expression of the conventional EV biomarkers known as tetraspanins, including CD9, CD81, and CD63, as well as the absence of lipoprotein contamination (Apolipoprotein B; APOB) (Figure 1D). The isolated adiposomes also demonstrated an expression of adipocytic proteins such as PPARγ, adiponectin, and fatty acid-binding protein 4 (FABP4) (Figure 1D), verifying their adipocytic origin.

### 3.3. Lipid Content in VAT Adiposomes

**Figure 2:**
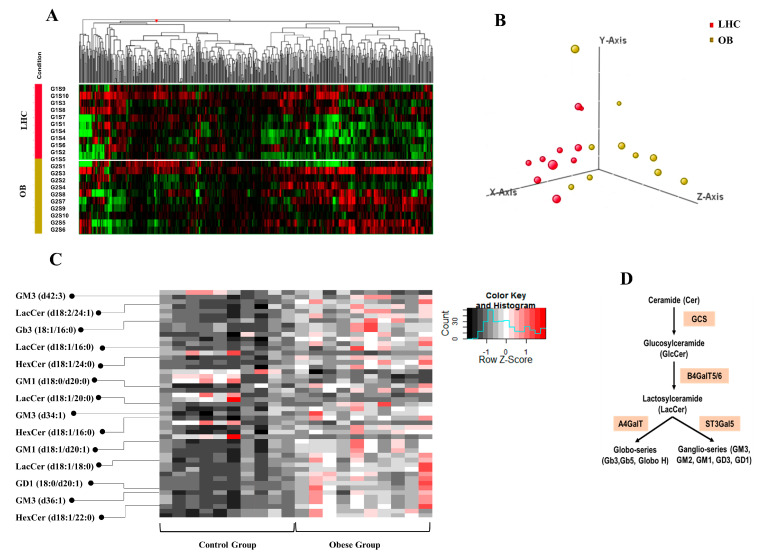
Lipid analysis in adiposomes isolated from OB-T2D and control subjects. (**A**) Heat map representation (log base 2 of normalized values) of 562 lipid species found in adiposomes isolated from OB-T2D and control subjects (*n* = 10, each). (**B**) Principal Component Analysis (PCA) of the identified lipids in isolated adiposomes. (**C**) Heat map representation of adiposomal lipids that were statistically different between OB-T2D and LHCs. The list next to the heat map is partial and includes only GSLs. (**D**) The pathway of ceramide metabolism into glycosphingolipids (GSLs).

A non-targeted mass spectrometry analysis was performed using the Q-TOF LC-MS system to analyze the relative abundance of different lipids in adiposomes isolated from VAT. Figure 2A shows a heat map representation (log base 2 of normalized values) of 562 lipid species that belong to 22 major lipid classes found in VAT adiposomes.

A scatterplot of the first two principal components demonstrates a reasonable separation of the OB-T2D and LHC phenotypes, implying that the lipid content of adiposomes is informative enough to distinguish the two groups (Figure 2B). Data, normalized to internal standards, showed significant variations in abundance of 59 lipid species between adiposomes isolated from OB-T2D subjects and LHCs as shown in the heatmap in Figure 2C. These lipid classes included glycosphingolipids (GLSs), ceramides, triglycerides, sphingomyelin, and Lysophosphatidylcholine. GSLs such as hexosyl ceramide (HexCer), lactosyl ceramide (LacCer), monosialodihexosylganglioside (GM3), and globotriaosylceramide (Gb3) were consistently detected in a higher amount in the OB-T2D group than LHCs and they were on the top of the differentially detected lipid species (Figure 2C). GSLs are generated via the glycosylation of ceramide. The attachment of glucose to ceramides via glucosylceramide synthase (GCS) yields GluCer, which can be converted to LacCer by the enzyme B4GalT. From LacCer, more complex GSLs, such as globosides and gangliosides, can be synthesized [40] (Figure 2D). Table 2 provides a summary of fold changes for the top GSLs that were differentially detected in VAT adiposome samples from OB-T2D patients in comparison with LHCs.

### 3.4. Adiposome Uptake by Endothelial Cells and Impact on Endothelial Cell Lipid Content

**Figure 3:**
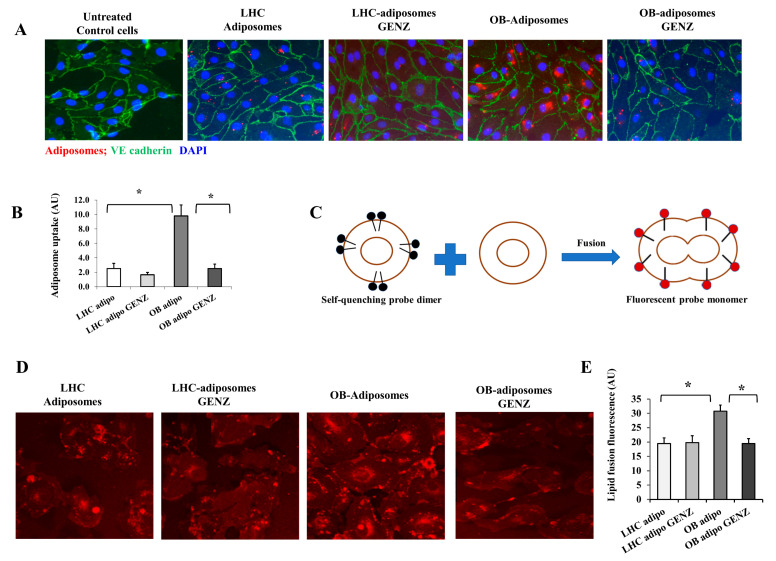
Adiposome uptake and fusion with endothelial cells. (**A**) Representative image of adiposome uptake (red fluorescent) by endothelial cells (cell membrane labeled green (VE-cadherin) and nuclei labeled blue (DAPI)). Adiposome GENZ refers to GSL-depleted adiposomes. (**B**) A quantification graph displaying the average ± SE of the red fluorescent signal intensity in (**A**) expressed in arbitrary units (AU). (**C**) A graphic illustration of the concept behind the rhodamine B chloride (R18) lipid fusion assay. (**D**) Confocal Microscopy images showing the fusion of R18-labeled adiposomes with endothelial cells. (**E**) A quantification graph displaying the average ± standard error of the red fluorescent signal intensity in (**D**) expressed in arbitrary units (AU). * *p*-value < 0.05.

To test endothelial cell uptake of adiposomes, cells were incubated for 30 min with fluorescently (BODIPY) labeled adiposome particles (8 × 10^9^). These adiposomes were isolated from VAT obtained from OB-T2D and LHC and cultured with and without the GluCer synthesis (GCS) inhibitor Genz-123346 to deplete the adiposome of GSLs. Cells were labeled with VE-cadherin to define cell boundaries, imaged using a confocal microscope, and the amount of red fluorescence was quantified using ImageJ software version 1.8.0. At corresponding time points, the attachment of OB-T2D adiposomes to endothelial cells was approximately 4-fold higher than LHC-adiposomes and was significantly reduced in GSL-depleted adiposomes (Figure 3A,B). To verify the fusion of adiposomes with endothelial cells, a lipid fusion assay was performed where adiposomes were labeled with the lipophilic probe, rhodamine B chloride (R18; red), and incubated for 30 min with HAMECs. This lipid-mixing assay is based on the self-quenching of R18 when the probe is incorporated into membranes (adiposomes) and the emission of red fluorescence when the fusion with target cell membranes (HAMECs) takes place (Figure 3C). The amount of red fluorescence in each treatment condition was quantified using ImageJ software version 1.8.0. At corresponding time points, the fusion of OB-T2D adiposomes to endothelial cells was ~60% higher than LHCs (*p* < 0.0001). This enhancement of lipid diffusion was not observed in response to GSL-depleted adiposomes (Figure 3D,E).

**Figure 4:**
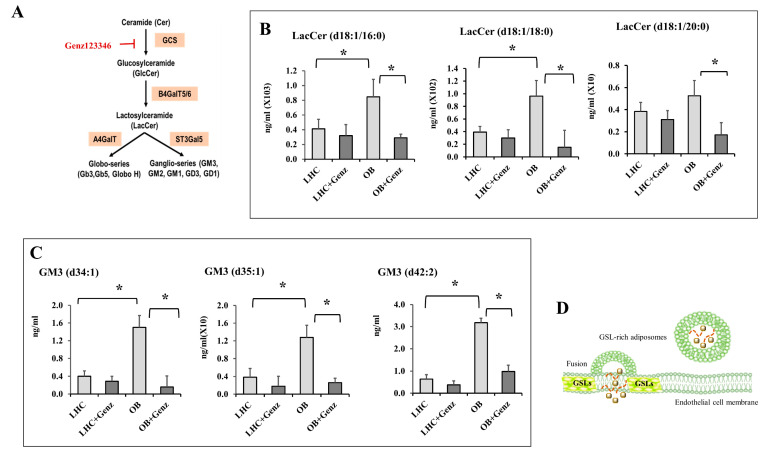
Lipids analysis in endothelial cell membrane after incubation with adiposomes. (**A**) Illustration of the pathway of ceramide metabolism into glycosphingolipids (GSLs) and the use of the GCS inhibitor, GENZ 123346. (**B**,**C**) represent targeted lipidomics analyses of LacCer and GM3, respectively, in endothelial cells in response to different treatment conditions (T2D-OB vs. LHC adiposomes with and without GSL depletion). (**D**) A depiction of the hypothesized fusion between GSL-rich adiposomes and endothelial cell lipid membranes, followed by the incorporation of GLSs into endothelial cell membranes. * p-value < 0.05.

To further investigate the effect of adiposome–endothelial cell fusion on the endothelial lipid content, targeted quantitative lipid analyses for LacCer and GM3 were performed in the extracted membranous fraction of endothelial cells using a QTRAP 6500 triple quadrupole mass spectrometer. For these experiments, adiposomes isolated from OB-T2D and LHCs were used with and without GSL depletion, which was accomplished by Genz123346 that inhibits the glucosylceramide synthase (GCS) and subsequently inhibits the formation of other GSLs (Figure 4A). HAMECs treated with OB-T2D adiposomes were found to have significantly higher concentrations (1 to 1.5-fold higher) of LacCer (d18:1/16:0) and LacCer (d18:1/18:0) (Figure 4B). Similarly, the GM3 lipid species, (d34:1), (d35:1), and (d42:2), were 2.4 to 4-fold higher in endothelial cells treated with OB-T2D adiposomes compared with those from LHCs (Figure 4C). These differences were not observed in GSL-depleted adiposomes. Collectively, these data suggest that GSL-rich adiposomes from OB-T2D patients fuse with endothelial cell lipid membranes and incorporate their GSLs into endothelial cell membranes, as illustrated in Figure 4D. It is conceivable that other biological materials carried by adiposomes, such as proteins, genetic materials, and other lipid species, may also be transferred to endothelial cells. However, the focus of the current investigation is on the GSL cargo and its possible role in aberrant signaling and endothelial dysfunction.

### 3.5. The Effect of Adiposomes on Endothelial Caveolae and Related Signaling

**Figure 5:**
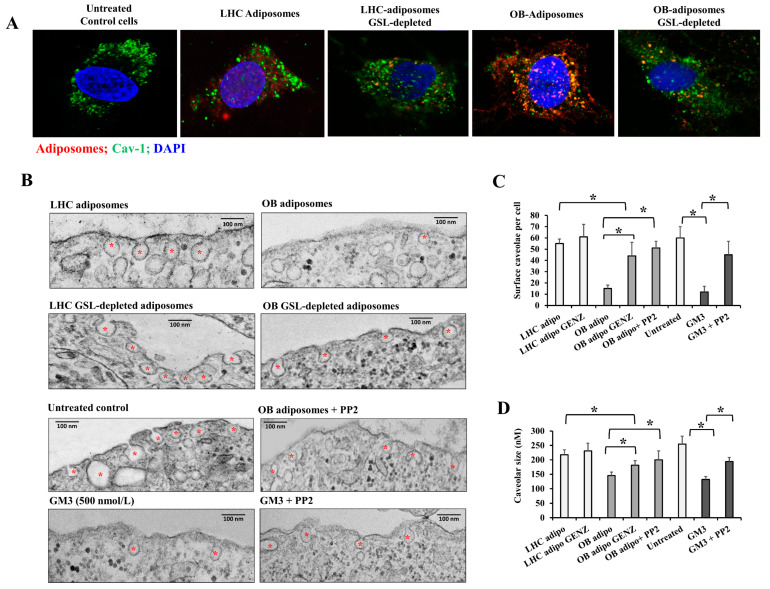
The effect of human adiposomes on endothelial cell caveolae. (**A**) Confocal Microscopy images depicting the colocalization of endothelial cav-1 protein (labeled green) and exogenously added human adiposomes (labeled red). (**B**) Transmission Electron Microscopy (TEM) of endothelial cells showing the status of surface caveolae under different treatment conditions. Surface caveolae are indicated by red asterisks. (**C**,**D**) Quantification charts showing the average number and size of caveolae in endothelial cells, respectively, in response to different treatments. * *p*-value < 0.05.

Caveolae are plasma membrane invaginations abundant in endothelial cells; they concentrate cell surface lipids and proteins involved in signal transduction and the regulation of endothelial cell function [21]. Our findings demonstrated a higher tendency for adiposomes to accumulate in endothelial cell surface caveolae. Figure 5A shows the colocalization of the red-labeled adiposomes with the green-labeled caveolin-1, the major protein in the caveolar structure. This colocalization (orange color) was more prominent in OB-T2D adiposomes than in LHC adiposomes, possibly owing to a higher affinity of GSL-rich adiposomes for caveolae. The adiposome-caveolae colocalization was diminished in GSL-depleted adiposomes, suggesting that GSLs play an important role in this phenomenon (Figure 5A).

Caveolae are dynamic structures, and their lipid content regulates the balance between their surface connection and scission. To test the impact of adiposomes on caveolar stability, electron microscopy images were taken for endothelial cells treated with adiposomes from OB-T2D patients and LHCs with and without inhibition of GSL synthesis (GSL-depleted adiposomes). Caveolae appear as flask-shaped invaginations (60–100 nm) located on the surface of endothelial cells (Figure 5B). Adiposomes obtained from OB-T2D subjects caused a significant detachment of endothelial cell surface caveolae and reduction in their size compared with those from LHCs (−72% and −33%, respectively, Figure 5C,D). It was also found that GSL depletion substantially negated these effects, subsequently maintaining the cell surface position and an average size of endothelial cell caveolae (Figure 5B–D).

Our research team previously reported the role of the caveolae-tethered Src kinase in caveolar detachment from the endothelial cell surface via inducing cav-1 phosphorylation. In addition, previous studies have shown that Src kinase acts as a lipid sensor that forms microdomains with GSLs on cell membranes [41]. Consequently, we conducted a set of experiments in which Src kinase activity in endothelial cells was inhibited using the specific inhibitor PP2 before adiposome administration. Indeed, our data demonstrated that Src kinase inhibition preserved the surface localization and size of endothelial cell surface caveolae to a significant degree (Figure 5B–D). Interestingly, treating endothelial cells with purified GM3 (500 nmol/L) decreased surface caveolae and their size to a level comparable to that induced by OB-T2D adiposomes, suggesting that the GSL content in OB-T2D adiposomes may be a major contributor to the observed alterations in endothelial cell caveolae.

**Figure 6:**
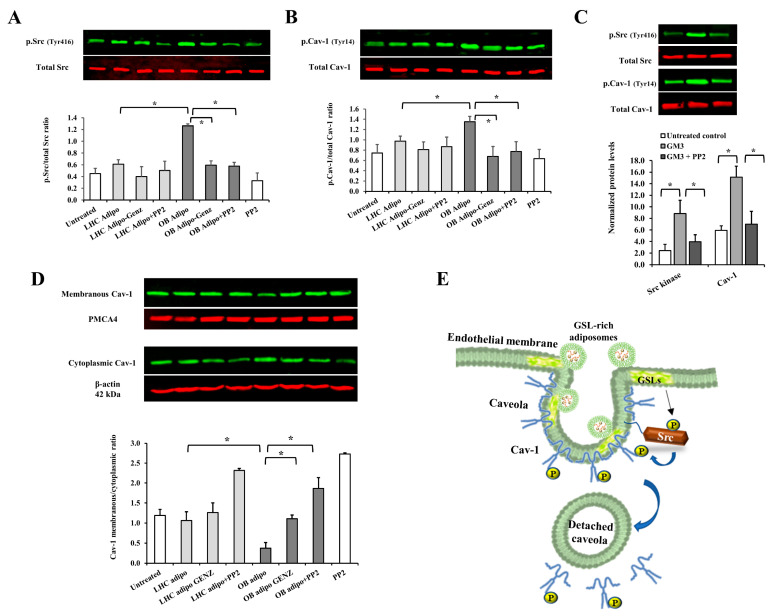
The effect of adiposomes on endothelial caveola-related signaling. (**A**–**C**) Western blots for p.Src and P.Cav-1 normalized to their total protein in endothelial cells treated with adiposomes from OB-T2D and LHCs (**A**,**B**) or GM3 (**C**) with and without PP2. Quantification charts display the means ± standard error of the signal intensity of the phosphorylated protein normalized to the corresponding total protein. (**D**) Western blots for membranous and cytoplasmic cav-1 protein in endothelial cells treated with adiposomes from OB-T2D and LHCs with and without PP2. The chart displays the means ± SE of the quantified membranous to cytoplasmic cav-1 ratio. (**E**) A diagram depicting the hypothesized effects of GSL-rich adiposomes on caveola-tethered proteins before caveolar detachments. * *p*-value < 0.05.

To further investigate this mechanism, the phosphorylated fraction of Src kinase (Tyr416) and cav-1 (Tyr14) was assessed in endothelial cells following incubation with adiposomes. Our findings indicated that OB-T2D adiposomes significantly induced Src phosphorylation (~3 folds) and cav-1 phosphorylation (82%) compared with adiposomes from LHCs (Figure 6A,B). Similar to other outcomes that were inhibited by GSL depletion from adiposomes, the induction of Src kinase and cav-1 phosphorylation was not observed in response to GSL-depleted adiposomes. In a subset of experiments, we verified the ability of GM3 (using 500 nmol/L) to promote Src kinase and cav-1 phosphorylation in endothelial cells, an effect that was suppressed by Src kinase inhibition (Figure 6C). To test whether caveolar loss from cell surface is due to caveolar fission and cellular internalization, we compared the membranous versus cytoplasmic protein fractions of cav-1 in treated endothelial cells. As predicted, the ratio of membrane-to-cytoplasmic cav-1 protein in cells treated with OB-T2D adiposomes was significantly reduced. GSL depletion from adiposomes and prior Src kinase inhibition both maintained this ratio in OB-T2D adiposome-treated cells (Figure 6D).

Together, these data suggest that GSL-rich adiposomes are capable of fusing with endothelial cell membranes with a higher affinity to the caveolar structure. These adiposomes activate Caveola-tethered proteins, including Src kinase and cav-1, whose phosphorylation promotes the loss of caveolar protein’s connection to the cell membrane (Figure 6E). This perturbation of EC caveolar structure is conceivably predicted to disrupt endothelial cell signaling and function. Consequently, we investigated the functional consequences of treating endothelial cells with OB-T2D, GSL-rich adiposomes in the subsequent sections.

### 3.6. The Impact of Adiposomes on eNOS Activity and Cav-1 Stability

**Figure 7:**
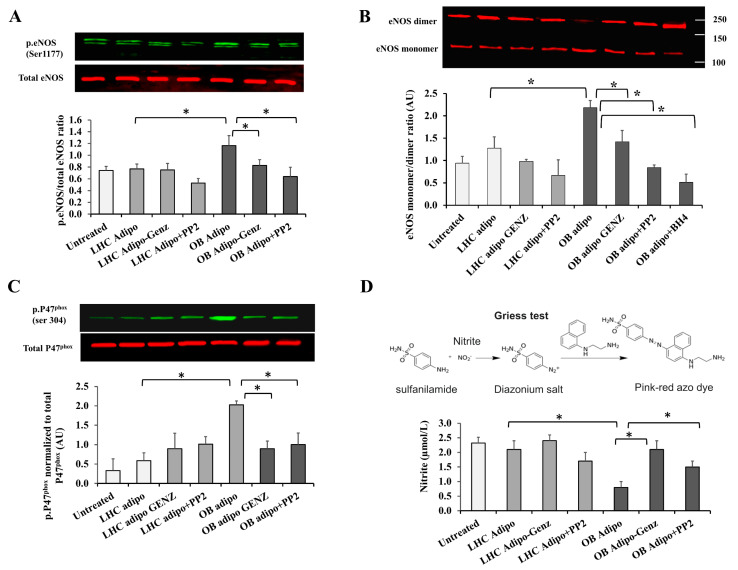
The effect of adiposomes on eNOS phosphorylation and nitric oxide production. Western blot images and quantification charts for p.eNOS normalized to its total protein (**A**), eNOS monomer to dimer ratio (**B**), and p.P47^phox^ normalized to its total protein (**C**) in endothelial cells treated with adiposomes from OB-T2D and LHCs (Native and GSL depleted) with and without PP2 or the cofactor BH_4_. (**D**) measurements of nitric oxide (NO) production using the Griess test, an analytical chemistry test that measures nitrates and nitrites via a series of reactions where nitrites are eventually converted to a colored Azo compound that was measured at 540 nm wavelength by a multimode plate reader. * *p*-value < 0.05.

Previous studies reported that eNOS activity is regulated by a physical interaction between eNOS and the scaffolding domain of cav-1. Accordingly, the absence of surface caveolae is anticipated to promote eNOS dysregulation and, consequently, endothelial dysfunction. To determine the effects of enhanced caveolar fission in response to adiposome fusion on eNOS status, we evaluated the phosphorylation of eNOS (ser 1177; induces eNOS activity) and its uncoupling (measured via estimating monomer to dimer ratio as we previously published [42]). In endothelial cells treated with OB-T2D adiposomes, the phosphorylated fraction of eNOS increased by 53% compared with cells treated with LHC adiposomes. The latter did not show any significant differences from the untreated control. GSL depletion in OB-T2D adiposomes normalized the levels of phosphorylated eNOS to those of the control. Similarly, inhibiting the Src kinase activity via PP2 reduced eNOS phosphorylation in cells treated with OB-T2D adiposome (Figure 7A).

Moreover, our data showed a significant increase in eNOS uncoupling in response to OB-T2D adiposomes, indicated by a 2-fold increase in eNOS monomer to dimer ratio compared with the untreated control. This effect was not observed in response to LHC adiposomes or GSL-depleted adiposomes; it was also abolished in cells where Src kinase activity was inhibited by PP2 and cells supplemented with the eNOS cofactor, BH_4_ (10^−5^ mol/L) (Figure 7B).

It has been previously shown that eNOS uncoupling in endothelial cells is augmented in the presence of superoxide radicals (O_2_^−^) produced by the enzyme NADPH oxidase isoform 2 (Nox2). The activity of Nox2 is mediated via the phosphorylation of its regulatory subunit p47^phox^ [43]. Therefore, in the current study, we measured the phosphorylated fraction of P47^phox^ (ser304) in response to adiposome treatment. Our data showed that OB-T2D adiposomes substantially induced P47^phox^ phosphorylation compared with untreated control (5-fold, *p* < 0.0001). However, these changes were significantly minimized by depleting adiposomes of GSLs (via GENZ 123346) or inhibiting Src kinase activity in endothelial cells via PP2 (Figure 7C).

To better understand the functional outcomes of these changes in eNOS phosphorylation, nitric oxide (NO) production was measured using the Griess test. The latter is an analytical chemistry test that measures the level of the NO metabolites, nitrates, and nitrites via a series of reactions where nitrites are eventually converted to a colored Azo compound that could be quantified via a plate reader (Figure 7D). In cells treated with OB-T2D adiposomes, NO was ~65% (*p* = 0.001) lower than those treated with LHC adiposomes. No significant differences in NO generation were observed between untreated cells and cells treated with LHC adiposomes. GSL depletion and Src kinase inhibition rescued NO generation in cells treated with OB-T2D adiposomes (1.6-fold and 88%, respectively, *p* < 0.0001).

**Figure 8:**
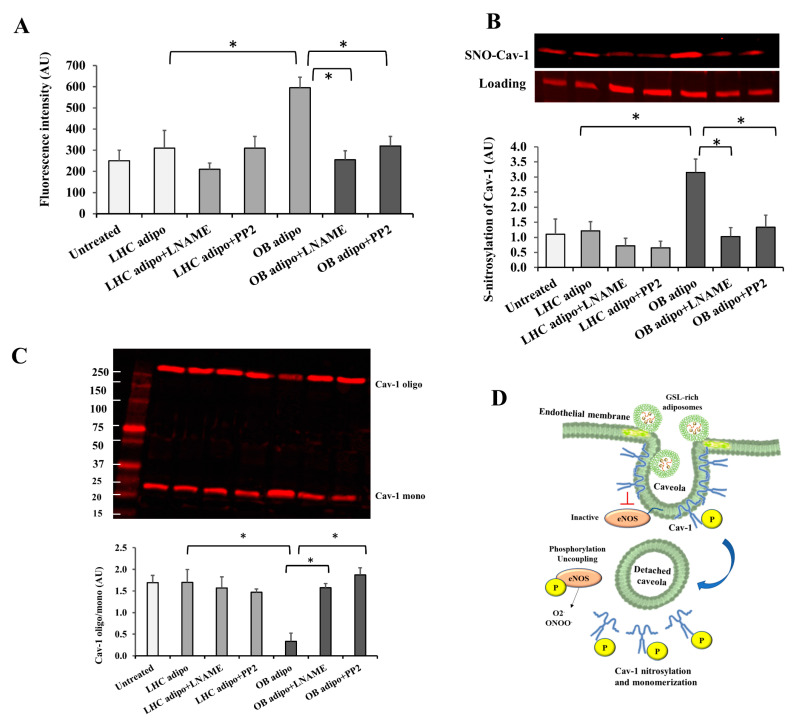
The effect of adiposomes on cav-1 stability. (**A**) peroxynitrite measurements in endothelial cells treated with adiposomes in the presence or absence of PP2 and LNAME. (**B**,**C**) Western blot images and quantification charts for S-nitrosylated cav-1 (**B**) and cav-1 oligomer to monomer ratio (**C**) in endothelial cells treated with adiposomes in the presence or absence of PP2 and LNAME. (**D**) A diagram illustrating the hypothesized effects of GSL-rich adiposomes starting with caveolar detachment and eNOS uncoupling, then leading to an augmented generation of superoxide and peroxynitrite and ultimately resulting in cav-1 nitrosylation and monomerization. * *p*-value < 0.05.

Previous studies have shown that uncoupled eNOS is itself a source of reactive O_2_ species (ROS), especially superoxide (O_2_^−^) that binds NO-forming peroxynitrites. Indeed, when measured in endothelial cells treated with OB-T2D adiposomes, peroxynitrite was found to be 1.4-fold higher than untreated cells. This effect was prevented by the eNOS inhibitor L-NAME (10^−4^ mol/L) or the Src kinase inhibitor PP2 (Figure 8A). No significant effects were observed in response to conditions where LHC adiposomes were added. Previously, we demonstrated that eNOS uncoupling in endothelial cells induced the formation of nitrotyrosine, which was reversed by LNAME [42]. Here, we examined whether eNOS uncoupling in HAMECs promotes cav-1 S-nitrosylation and whether this is accompanied by disruptions in cav-1 oligomerization. Indeed, our findings showed a 3-fold increase in cav-1 S-nitrosylation upon incubation with OB-T2D adiposomes (Figure 8B). This effect was reduced significantly and normalized to the untreated control level by L-NAME or PP2, suggesting Src-dependent eNOS regulation upstream of cav-1 S-nitrosylation. Cav-1 protein exists in monomeric and oligomeric forms; the latter promotes the self-assembly of caveolae [44]. Accordingly, the ratio of cav-1 oligomers to monomers is indicative of caveolar assembly and stability. Here, we assessed changes in the cav-1 oligomer/monomer ratio under different treatment conditions, as shown in Figure 8C. A remarkable decrease (~5-fold) in the oligomer/monomer ratio was observed in response to OB-T2D adiposomes, and this was entirely reversed by pretreatment with L-NAME or PP2 (Figure 8C). Neither L-NAME nor PP2 influenced the cav-1 oligomer/monomer ratio in cells treated with LHC adiposomes.

Taken together, these findings suggest that cav-1 S-nitrosylation and monomerization in response to OB-T2D adiposome treatment are dependent on eNOS and Src kinase activity. As depicted in Figure 8D, it is anticipated that GSL-rich adiposomes will activate Src kinase, which is known to be anchored to endothelial cell surface caveolae, followed by activation of cav-1 and dissociation of cell surface caveolae. Consequently, caveolar fission will liberate eNOS from inhibition, resulting in increased eNOS phosphorylation and uncoupling. Eventually, excessively produced superoxide and peroxynitrites can induce S-nitrosylation of cav-1, inhibiting its oligomerization and caveolar structure assembly.

### 3.7. The Impact of Adiposomes on Shear Stress Response and Albumin Uptake in Endothelial Cells

Endothelial cells are continuously exposed to hemodynamic forces due to the flowing blood in vessels. These forces cause cyclic stretches of endothelial cells, which can be converted into intracellular signaling that regulates several cellular functions such as permeability, angiogenesis, migration, and several others. Shear stress and cyclic strain are perceived by endothelial cells as mechanical stimuli that induce several responses, including changes in cell morphology, cell function, and gene expression. Therefore, any impairments in endothelial cell responses to shear stress result in endothelial dysfunction and predispose to cardiovascular diseases. Caveolae have been found to contribute significantly to endothelial cell response to shear stress and the ability of cells to stretch, align with the stream, and translate mechanical stimuli to appropriate cellular responses.

**Figure 9:**
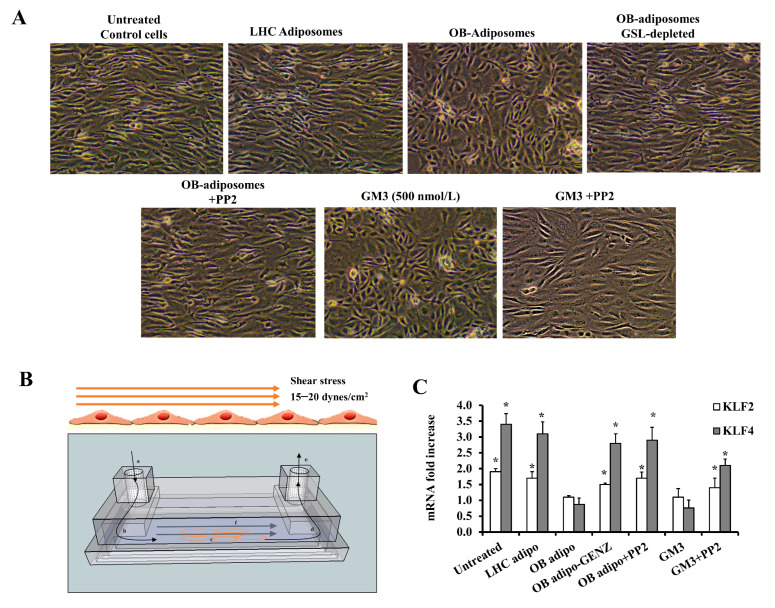
The effect of adiposomes on endothelial cell shear stress response. (**A**) Bright-field images of adiposome or GM3-preconditioned endothelial cells exposed to shear stress (15–20 dynes/cm^2^) for 2–4 h. (**B**) A graphic illustration of the chamber slide used to expose endothelial cells to shear stress (a, inlet port; b, inlet flow chamber; c, cells; d, outlet flow chamber; e, outlet port; f, laminar flow). (**C**) Fold changes in normalized mRNA expression of KLF2 and KLF4 genes in response to shear stress compared with the baseline. * *p*-value < 0.05.

We showed above that OB-T2D adiposomes caused caveolar fission and loss from the endothelial cell surface. Therefore, we sought to test the effect of adiposomes on endothelial cell response to shear stress (SS) by culturing cells in parallel chamber slides under a shear rate of 15 to 20 dynes/cm^2^ for 2 to 4 h. The flowing media in the microfluidics system contained the designated treatment (adiposomes or GM3 with and without PP2). Figure 9A shows cells elongated and aligned under SS, consistent with reported changes by others [45,46]. Endothelial cells preconditioned with OB-T2D adiposomes or GM3 were irregularly oriented and not aligned with the shear direction (Figure 9A), indicating a lack of response to SS. This response was rescued by Src kinase inhibition via PP2, suggesting a mediating role of Src kinase in this adiposome-induced phenotype. On the other hand, cells treated with LHC adiposomes or GSL-depleted adiposomes did not show any significant changes compared with the untreated control.

The cartoon illustration in Figure 9B is meant to depict the concept of endothelial cells cultured in chamber slides and aligned in the direction of SS. The bar chart in Figure 9C represents the fold change in mRNA expression levels of two of the most prevalent SS-responsive genes, Krüppel-like Factor 2 (KLF2) and KLF4, compared with the baseline (prior to SS). The mRNA levels of these genes exhibited a significant rise in response to SS across all treatment conditions, except for the OB-T2D and GM3 treatments. However, this induction was reinstated upon depletion of GSL and inhibition of Src kinase. The findings of this study indicate that adiposomes rich in GSLs play a significant role in inhibiting the response of endothelial cells to SS, interfering with a crucial physiological process that regulates endothelial cell function.

**Figure 10:**
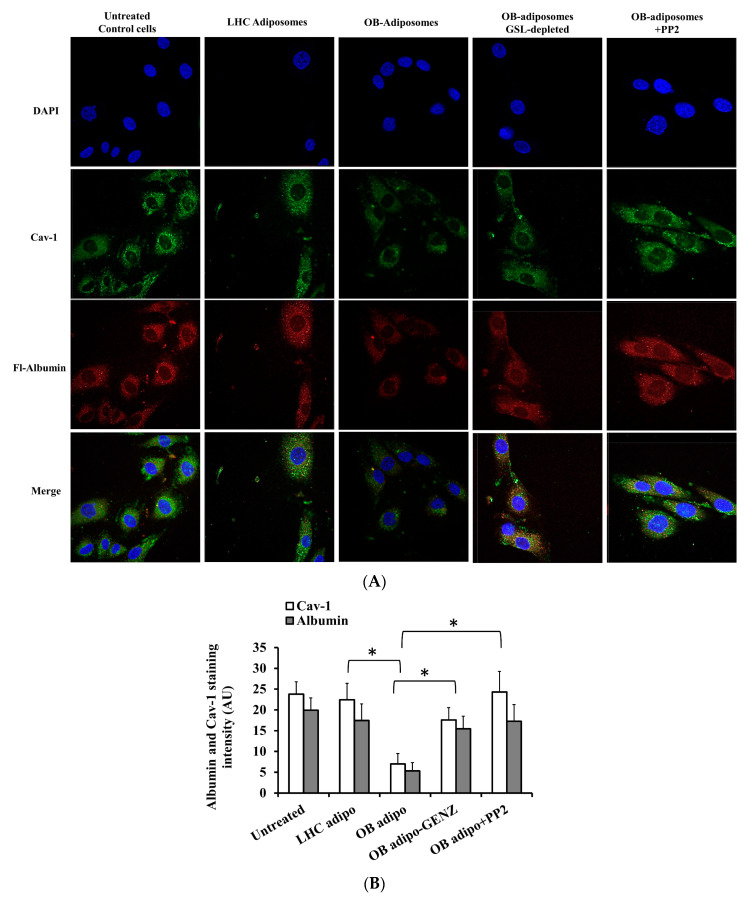
The effect of adiposomes on albumin uptake by endothelial cells. (**A**) Representative images of the colocalization of fluorescent albumin (red) and cav-1 (grees) in endothelial cells untreated, treated with LHC adiposomes, and treated with OB-T2D adiposomes with and without GSL depletion or PP2. (**B**) A quantification chart showing the means ± standard error of the fluorescent signal intensity expressed in arbitrary units (AUs). * *p*-value < 0.05.

We next tested the effect of adiposomes on albumin uptake by endothelial cells (Figure 10A). Cells preconditioned with adiposomes with or without PP2 were incubated with Texas Red conjugated BSA for 30 min and then fixed and stained with cav-1 and DAPI and imaged with Confocal Microscopy. The adiposomes derived from LHC did not elicit any observable alterations when compared with the untreated control group. Conversely, the adiposomes derived from OB-T2D exhibited a notable decrease in albumin intake and a drop in cav-1 staining (3.3-fold, *p* < 0.001, Figure 10B). These modifications were diminished in GSL-depleted OB-T2D adiposomes, in which albumin uptake and cav-1 expression increased substantially. Similarly, the inhibition of Src kinase activity in endothelial cells not only restored the expression of cav-1 but also reinstated the uptake of BSA to levels that were comparable to those observed in the untreated control (Figure 10A,B). These findings suggest that Src kinase plays a substantial role in the loss of caveolae and the subsequent decline in albumin uptake.

### 3.8. Effects of Adiposomes on Vascular Function

**Figure 11:**
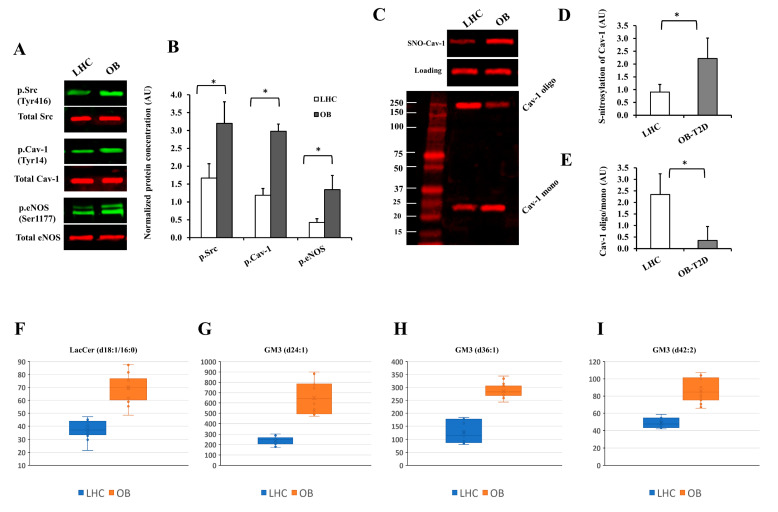
Src kinase, cav-1, and eNOS protein status in human AT biopsies. (**A**,**B**) Western blot images and quantification charts of the phosphorylated fraction of Src kinase, cav-1, and eNOS normalized to their total protein in VAT biopsies from OB-T2D patients (*n* = 15) and LHCs (*n* = 15). (**C**–**E**) Western blot images and quantification charts of the nitrosylated cav-1 and cav-1 oligomer to monomer ratio in VAT biopsies from OB-T2D patients (*n* = 15) and LHCs (*n* = 15). (**F**–**I**) Targeted lipidomics analyses of LacCer and GM3 in human AT-isolated endothelial cells. * *p*-value < 0.05.

We sought to determine whether human AT-isolated endothelial cells exhibited changes analogous to those observed in vitro in endothelial cells treated with OB-T2D adiposomes. To this end, CD31-positive endothelial cells isolated from AT samples from OB-T2D and LHC were analyzed for protein expression and GSL content using Western blotting and mass spectrometry, respectively. Indeed, our results demonstrated significantly higher levels of phosphorylated Src kinase (1-fold), cav-1 (1.5-fold), and eNOS proteins (2-fold) in endothelial cells isolated from OB-T2D subjects compared with LHCs (Figure 11A,B). In addition, cav-1 S-nitrosylation was 1.5-fold higher, and the cav-1 oligomer/monomer ratio was 4.7-fold lower in the OB-T2D subjects compared with LHC adiposomes (Figure 11C–E). Targeted lipidomic assays showed significant variations in four GSL species, namely LacCer (d18:1/16:0), GM3 (d24:1), GM3 (d36:1), and GM3 (d42:2). The average levels of these species in OB-T2D patients were found to be 85%, 173%, 125%, and 78% higher compared with those in LHCs, respectively (Figure 11F–I).

**Figure 12:**
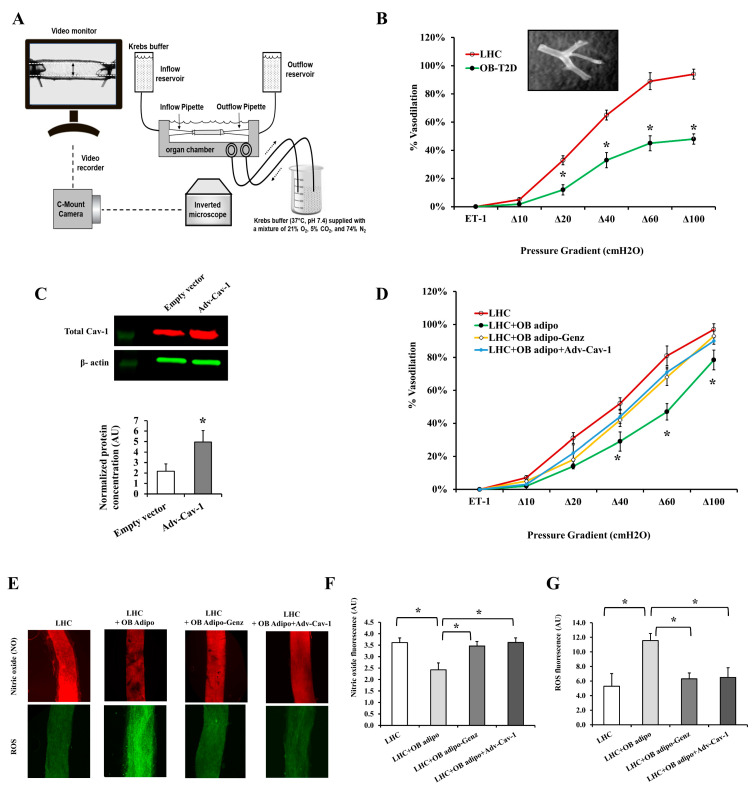
The effect of adiposomes on vascular function. (**A**) A graphic illustration of the flow-induced dilation (FID) measuring setup. (**B**) Graphic representation of FID measured in VAT arterioles from OB-T2D patients (*n* = 15) and LHCs (*n* = 15). (**C**) Western blot and quantification chart for total cav-1 in arterioles transfected with an empty vector versus Adv-cav-1. (**D**) FID measurements in LHC arterioles exposed to OB-T2D adiposomes (±GSL depletion) in the presence and absence of Adv-cav-1. (**E**–**G**) representative images and quantification charts for nitric oxide (red fluorescence) and ROS (green fluorescence) production in LHC arterioles under different exposure conditions. AU (Arbitrary units). * *p*-value < 0.05.

GSLs have been associated with disturbed vascular biology and all events contributing to atherosclerosis, such as inflammation, oxidative stress, plaque formation, and smooth muscle cell proliferation [16]. Our research team used a well-established technique for quantifying flow-induced dilation (FID) in arterioles that have been extracted from AT, cannulated in organ chambers, and exposed to a progressively increasing intraluminal pressure gradient, as depicted in Figure 12A. Using this approach, we previously reported impaired FID in OB subjects compared with LHCs [36], and our current data reproduce these findings in AT arterioles obtained from OB-T2D and LHC subjects (Figure 12B, * *p* < 0.05). FID at ∆60 cmH_2_O, which reflects physiological arteriolar pressure, was 65% lower in OB-T2D than in LHCs. Incubation of the isolated arterioles from LHCs with adiposomes (2 × 10^11^ particles) obtained from the AT of OB-T2D subjects for 4 h impaired the FID by 60% compared with untreated vessels (* *p* < 0.05); this reduction was not significant in arterioles treated with GSL-depleted adiposomes (Figure 12D). Also, rescuing the production of cav-1 using Ad-cav-1 transfection considerably enhanced the protein level of cav-1 (Figure 12C), leading to an improvement in arteriolar FID in LHC arterioles that were treated with OB-T2D adiposomes (Figure 12D).

Our results provide additional evidence of a substantial increase in the arteriolar production of ROS and the reduction in nitric oxide (NO) generation following incubation with OB-T2D adiposomes. This effect was shown to be suppressed when the adiposomes were depleted of GSLs, as well as when cav-1 was restored through the transfection of Ad-cav-1 (Figure 12E–G). These findings provide evidence that supports the proposed negative impact of GSL-rich adiposomes on microvascular function. Additionally, the study identifies NO and ROS as potential targets within this pathway.

## 4. Discussion

The primary findings of the present study indicate that adiposomes derived from individuals with obesity and type 2 diabetes (OB-T2D) contain higher levels of glycosphingolipids (GSLs) compared with those from lean, healthy controls. These GSLs are believed to contribute, at least in part, to the detrimental effects of OB-T2D adiposomes on endothelial cells. Furthermore, when OB-T2D adiposomes were introduced to endothelial cells, they induced detachment of cell surface caveolae through the activation of Src kinase and phosphorylation of cav-1. This effect had a negative impact on endothelial nitric oxide synthase (eNOS) function, resulting in eNOS uncoupling, reduced nitric oxide (NO) production, and increased generation of peroxynitrites. These molecular changes were also reflected in the impaired functionality of endothelial cells, characterized by a loss of response to shear stress and reduced intake of albumin. Finally, the adiposomes of OB-T2D were found to have a detrimental effect on flow-induced dilation (FID) and nitric oxide (NO) production in healthy arterioles.

The endocrine roles of AT have primarily been ascribed to adipokines, a diverse group of soluble bioactive molecules that are released from adipocytes, including but not limited to leptin and adiponectin [47]. Relatively recently, adipocyte-derived extracellular vesicles (adiposomes) have been discovered [48]. These vesicles include a wide range of lipids, proteins, and genetic material, suggesting an additional mechanism for endocrine signaling originating from AT. Dysfunctional adipocytes with dysregulated metabolism in obese and diabetic AT may release adiposomes with an altered cargo of lipids and other biological content. This content may promote vascular dysfunction upon interaction with endothelial cells. Therefore, comprehensive studies that explore the potential role of adiposomes in predisposing obese, diabetic individuals to cardiovascular diseases (CVD) are required.

The association between dysregulated lipid metabolism in AT and cardiovascular risk has been established [49]. However, the mechanism by which the dysregulated lipid milieu is transported to vascular tissues has primarily been attributed to soluble factors, neglecting the potential role of adiposomes. A significant portion of secretions originating from AT are encapsulated within adiposomes, and prior research has indicated that this method of packaging helps protect the cargo and improves its chance of reaching distant cells and tissues [50]. However, due to technical difficulties in isolating and characterizing human adiposomes, our understanding of their mechanistic contribution to CVD remains limited to findings from few in vitro studies [51].

It is worth mentioning that one-third of the extracellular vesicle (EV) volume is lipids [52]; however, unlike the protein and nucleic acid cargo, the lipid content in EVs has been far less explored, and its implication in vascular function has not been investigated. In the current study, we analyzed the differences in the lipid content of adiposomes between OB-T2D patients and lean, healthy controls (LHCs). Our findings demonstrated significantly higher levels of GSLs in the OB-T2D adiposomes, on top of which were lactosylceramide and GM3. GSLs are generated via the glycosylation of ceramide. The latter is a bioactive sphingolipid, and its generation in inflamed tissues is mainly mediated by sphingomyelinase (SMase) [53], which increases in chronic inflammatory illnesses such as obesity, diabetes, and CVD [14,15]. Glycosylation of ceramides is upregulated in diabetic patients and is driven by the local milieu of high glucose and inflammation-mediated induction of enzyme activity [12,13]. The attachment of glucose to ceramide via glucosylceramide synthase (GCS) yields glucosylceramide (GluCer), which can be converted to lactosylceramide (LacCer) by the enzyme glucosylceramide β4-galactosyltransferase (B4GalT). From LacCer, more complex GSLs, such as globosides and gangliosides, can be synthesized via different sialyltransferases and galactosyltransferases [40].

GSLs serve as secondary messengers in various signaling pathways and are implicated in the pathological consequences of T2D, such as retinopathy, neuropathy, atherosclerosis, and coronary artery diseases [16,17,18]. However, the critical barrier to progress in this area is that the precise mechanism that mediates GSL impact on vascular function is poorly understood. These GSLs are glycosylated lipids that were shown to be induced under conditions of hyperglycemia and inflammation. So, indeed, adiposomes reflect the internal environment and metabolic status of dysfunctional AT, which lends significance to the investigation of the biological messages that are carried and transferred by adiposomes to other cells, such as endothelial cells.

In order to gain insight into the impact of adiposomes on endothelial cells, we conducted the current investigation to assess the structural and functional consequences observed in endothelial cells following treatment with adiposomes. Compared with healthy adiposomes, GSL-rich adiposomes from OB-T2D patients were able to more efficiently incorporate GSLs such as lacCer and GM3 into the endothelial cell membrane. In addition, these GSL-rich adiposomes tended to accumulate in caveolae-rich regions, which led to their detachment from the cell surface or diminution in size. Previous studies have shown that GSLs such as LacCer are internalized by a caveolae-associated mechanism [54]. This finding may explain the observed propensity of GSLs to accumulate in locations abundant in caveolae.

Even though caveolae are typically associated with lipids on the cell surface, they undergo short-range cycles of fission and fusion, and their biogenesis and stability on the cell surface are dependent on the tightly regulated balance of their lipid composition. Hubert et al. [20] demonstrated that synthetic GSL-rich fusogenic liposomes reduced the diameter of caveolar necks in HeLa cells, leading to caveolar fission and confirming the fact that caveolar stability is highly sensitive to changes in the plasma membrane lipid composition. However, no comparable research on endothelial cells has been conducted. Consequently, we believe that our study is one of the first to examine the effect of GSL-rich adiposomes isolated from OB-T2D patients on the lipid content of endothelial membranes and caveolar fission. The lack of effect of GSL-depleted adiposomes further supported the role of GSLs in inducing these effects.

Inhibition of glucosylceramide synthase (GCS) in LHC adiposomes did not have the same significant impact on GSL levels as in OB-T2D adiposomes. We believe this is due to the low baseline levels of GSLs in LHCs. Previous studies have documented the increased activity of the GCS enzyme under conditions of inflammation and hyperglycemia observed in obese diabetic patients, lending support to this assumption [12,55]. It can be deduced that the outcome of GCS inhibition is dependent on baseline levels of GSLs and GCS activity, a trait also observed in lipid-lowering drugs that do not substantially lower lipid levels in individuals with normal lipid profiles.

Cav-1 is an essential structural element of caveolae, and its phosphorylation by Src kinase is correlated with an increase in caveolae-mediated fission from the cell surface. We previously showed that cav-1 phosphorylation at tyr-14 induces the separation of adjacent negatively charged N-terminal phosphotyrosine residues. This event facilitated the swelling of caveolae structures, ultimately resulting in their detachment from the plasma membrane [56]. The present investigation revealed that adiposomes derived from OB-T2D individuals elicited a more pronounced activation of Src kinase and cav-1 phosphorylation compared with adiposomes obtained from healthy control subjects. A significant reduction in cav-1 phosphorylation and caveolar detachment was observed when Src kinase activity was suppressed in endothelial cells. These findings imply that the influence of adiposomes on cav-1 and caveolae is facilitated by the activation of Src kinase.

Caveolae are essential structures in endothelial cells, as they serve to concentrate a diverse array of transmembrane signaling molecules (signalosomes) that play a regulatory role in endothelial cell functions [21]. Caveolae serve as a scaffold for the inactive form of eNOS, ensuring the maintenance of well-regulated cycles of eNOS activation and inhibition and subsequently, proper vasoreactivity. Caveola-eNOS interactions are governed by stimuli such as shear stress, which results in eNOS dissociation from caveolae and subsequent phosphorylation and activation [57]. The loss or reduction in surface caveolae is anticipated to disrupt these tightly regulated cycles. In the present study, the adiposome-induced fission of caveolae from the endothelial cell surface was associated with an increase in eNOS phosphorylation. However, the induction of eNOS phosphorylation was not accompanied by an increase in nitric oxide production. Instead, peroxynitrate production was stimulated, indicating an increase in superoxides and other reactive oxygen species. Indeed, the data presented in this study clearly indicate notable elevations in NADPH oxidase (Nox2) activity and peroxynitrite generation, which were observed concurrently with the stimulation of eNOS phosphorylation. Moreover, eNOS monomerization increased in response to GSL-rich adiposomes from OB-T2D patients.

These observations collectively refer to a unique phenomenon known as eNOS uncoupling, in which eNOS produces highly reactive superoxide, which, in the presence of little NO, induces the production of the more reactive molecule, peroxynitrite [58]. However, it is unclear whether eNOS uncoupling is a consequence or cause of induced oxidative stress and where the process begins. Previous research uncovered discrepancies between eNOS phosphorylation and uncoupling, leading researchers to conclude that eNOS phosphorylation at ser1177 is not always accompanied by NO production [59]. According to our research, the induction of eNOS uncoupling by OB-T2D adiposomes was found to be disrupted by inhibitors of Src kinase, as well as by external replenishment with BH_4_. The latter is a necessary co-factor that facilitates eNOS activity and NO production from L-arginine. Oxidation of BH_4_ results in transmutation to BH_2_, which disturbs the normal electron flow and promotes superoxide formation, resulting in a vicious circle of induced oxidative stress [60].

The overproduction of reactive oxygen species (ROS), particularly peroxynitrites, plays a significant role in the promotion of S-nitrosylation of cellular proteins [61]. In the present investigation, we detected a notable augmentation in the S-nitrosylation levels of cav-1. Given that the assembly of cav-1 protein into caveolae necessitates oligomerization, our objective was to examine the oligomer-to-monomer ratio of this protein [62]. Our data demonstrated a decrease in this ratio, which is anticipated to be a consequential result of cav-1 S-nitrosylation. This notion is confirmed by prior research from our group and others indicating impairments in the capacity of cav-1 to oligomerize when it undergoes nitrosylation [63,64]. It is anticipated that these effects will impede the process of caveolar construction and exacerbate the deficiency of caveolae on the cell surface.

In addition to eNOS signaling, caveolae contain a variety of mechanosensitive molecules, including mechanosensitive ion channels, G-protein coupled receptors (GPCRS), extracellular signal-regulated kinases (ERK), receptor tyrosine kinases (RTK), and others [65,66,67]. These proteins convert shear stress into cell signaling through multiple mechanisms, including the induced expression of the transcription factors KLF2 and KLF4. These factors transcribe genes that modify endothelial cell morphology and physiology in response to shear stress [68]. In this investigation, the shear response of endothelial cells treated with OB-T2D adiposomes was found to be impaired. These impairments were observed at the morphological and molecular levels, as indicated by the lack of cell streaming and the absence of induced mRNA expression of the KLF2 and KLF4 transcription factors, respectively. These effects were not observed in response to GSL-depleted OB-T2D adiposomes and were eliminated by the Src kinase inhibitor, PP2. However, more in-depth research on the effect of adiposomes on shear stress-related signaling pathways is required.

In order to investigate additional functional implications of caveolar loss generated by obesity-related type 2 diabetes (OB-T2D), we analyzed albumin uptake. A substantial body of research supports the crucial role of endothelial cell caveolae in the effective uptake of albumin [69]. In fact, the current study demonstrated that GSL-rich adiposomes from OB-T2D patients decreased endothelial albumin uptake, corroborating the impairment of caveola-mediated endocytosis. The latter refers to a mechanism by which endothelial cells facilitate the absorption of several molecules, including insulin. Consequently, the disruption of this process is anticipated to result in many adverse outcomes. In addition, several drugs, including the chemotherapeutic agent paclitaxel, are administered in albumin-bound form [70]. Consequently, the inability of endothelial cells to absorb albumin will result in decreased bioavailability and bioefficacy of these medications. These findings have clinical relevance for the current investigation and may help explain the treatment resistance observed in obese diabetic patients in response to certain medications. Moreover, these findings pave the way for a new field of research in which adiposomes and their cargo could be examined as a potential cause of therapeutic resistance in certain diseases such as obesity and diabetes.

The observed alterations in the Src/cav-1/eNOS axis in cultured endothelial cells were confirmed in AT-isolated human endothelial cells. Compared with healthy controls, endothelial cells from OB-T2D patients demonstrated elevated levels of phosphorylated Src kinase, cav-1, and eNOS. In addition, the cav-1 protein in the endothelial cells of OB-T2D patients was significantly more S-nitrosylated and had a reduced oligomer-to-monomer ratio. Furthermore, targeted lipidomics analysis revealed increased levels of LacCer and GM3 species in OB-T2D patients’ endothelial cells compared with healthy controls. Notably, GM3 and LacCer exhibit distinct subspecies that differ with respect to their sphingosine base structure, fatty acid chain length, double bonds, and other related characteristics. Consequently, there is typically substantial variation among individuals, which explains the lack of an exact match between the LacCer and GM3 subspecies differentially detected in human endothelial cells and those observed in in vitro-cultured human endothelial cells. In conclusion, these findings provide support for the credibility of our in vitro experiments and suggest that the observed ex vivo impact of adiposomes on cultured endothelial cells closely mirrors their actions within the human body.

Arteriolar flow-induced dilation (FID) is a technique utilized by our group and others to test microvascular vasoreactivity in human arterioles promptly after their removal from the body. These arterioles are expected to be directly affected by the molecular changes in the AT. Also, these small arterioles are the primary site of peripheral resistance and blood pressure regulation, making these studies clinically relevant and highly impactful. Moreover, our team and others have refined the methodologies for manipulating gene expression in these arterioles, making them well-suited for conducting mechanistic studies [38]. In our previous studies, we demonstrated an impaired FID of AT arterioles isolated from obese or OB-T2D individuals compared with healthy counterparts [36]. In the current investigation, we observed FID impairments in healthy arterioles incubated with OB-T2D adiposomes, an effect not observed in response to GSL-depleted adiposomes and partially suppressed by cav-1 transfection (overexpression). To the best of our knowledge, this is the first study to test the effect of human-isolated adiposomes on arteriolar FID. These findings are novel, and we believe they pave the way for further mechanistic studies of the effect of adiposomes on arteriolar reactivity. Furthermore, these findings can point to adiposomal cargo as a potential mechanistic link between metabolic disorders and vascular dysfunction.

## 5. Conclusions

The current study establishes a new avenue for research into the complex cross-talk between dysfunctional adipocytes and endothelial cells. It is important to note, however, that this interaction is multifaceted and that other signaling molecules that we did not evaluate may be involved. It is also important to note that this research has limitations. First, while we focused on GSL cargo, we must acknowledge that other lipids, such as cholesterol, when introduced in excess to endothelial cells, may affect lipid raft structures such as caveolae. Moreover, it should be noted that although our study primarily investigates the impact of lipid cargo, it would be premature to assert that the observed influence of adiposomes on endothelial function can be only attributable to lipid contents. It is imperative to also take into account and examine other cargo components, such as proteins and miRNAs, in order to comprehensively understand the underlying mechanisms. Finally, further work is needed to explore the mechanisms by which endothelial cells ingest adiposomes, as well as whether this process involves endocytosis via caveolae or fusion with the membrane lipids.

## Figures and Tables

**Table 1:** Anthropometric and cardiometabolic characteristics of the participants.

Variable	LHC (*n* = 15, 7♀)	OB-T2D (*n* = 15, 8♀)	*p*-Value
Age, y	35 ± 5	34 ± 7	NS
**Anthropometric DEXA measurements**			
Weight, kg	75.5 ± 2.4	147.7 ± 19.4	0.0009
BMI, kg/m^2^	22.8 ± 1.6	46.3 ± 8.1	0.0082
**Metabolic and cardiovascular measurements**			
FPI, µU/mL	8.3 ± 0.3	14.4 ± 2.4	0.0176
FPG, mg/dL	91 ± 4	130 ± 12	0.0046
HOMA-IR	2.1 ± 0.2	5.3 ± 0.4	<0.0001
HbA1c, %	5.4 ± 0.2	6.7 ± 0.3	0.0012
Chol, mg/dL	155.5 ± 7.0	186.8 ± 12.0	0.0323
LDL, mg/dL	87.1 ± 6.0	104.5 ± 5.9	0.0480
HDL, mg/dL	55.0 ± 4.7	37.0 ± 6.7	0.0363
Trig, mg/dL	95.7 ± 4.5	131.0 ± 6.2	0.0001
HR, bpm	75 ± 4	84 ± 6	NS
SBP, mmHg	115 ± 2	124 ± 6	NS
DBP, mmHg	75 ± 2	84 ± 3	0.0187

BM: body mass index; Chol: cholesterol; DBP: diastolic blood pressure; FPG: fasting plasma glucose; FPI: fasting plasma insulin; HbA1c: glycosylated hemoglobin; HDL: high-density lipoprotein; HOMA-IR: homeostatic model assessment for insulin resistance; HR: heart rate; LDL: low-density lipoprotein; LHC: lean healthy controls; OB-T2D: obese type 2 diabetics; SBP: systolic blood pressure; Trig: triglycerides.

**Table 2:** Top differentially detected GSLs in VAT adiposomes from OB-T2D and LHCs.

Lipid Species	Fold Change	Q-Value *
Gb3 (18:1/16:0)	1.8	0.0005
GD1 (18:0/d20:1)	3.3	0.0032
GM1 (d18:0/d20:0)	2.0	0.0007
GM1 (d18:1/d20:1)	1.9	0.0021
GM3 (d34:1)	2.2	0.0015
GM3 (d36:1)	1.8	0.0038
GM3 (d42:3)	2.1	0.0008
HexCer (d18:1/16:0)	1.6	0.0014
HexCer (d18:1/22:0)	3.1	0.0039
HexCer (d18:1/24:0)	2.2	0.0008
LacCer (d18:1/16:0)	1.8	0.0002
LacCer (d18:1/18:0)	2.2	0.0029
LacCer (d18:1/20:0)	1.9	0.0009
LacCer (d18:2/24:1)	1.9	0.0004

* Q-value is the *p* value after correcting for multiple testing using Benjamini-Hochberg method.

## Data Availability

All results from the current study are available in the article.
